# Experimental cholesteatoma: a comparison between spontaneous and induced models

**DOI:** 10.1016/j.bjorl.2021.09.003

**Published:** 2021-10-25

**Authors:** Felipe da Costa Huve, Jefferson André Bauer, Fábio André Selaimen, Maurício Noschang Lopes da Silva, Sady Selaimen da Costa

**Affiliations:** a Hospital de Clínicas de Porto Alegre, Departamento de Otorrinolaringologia - Cirurgia de Cabeça e Pescoço, Porto Alegre, RS, Brazil; b Universidade Federal do Rio Grande do Sul, Porto Alegre, RS, Brazil

**Keywords:** Cholesteatoma, Pathogenesis, Mongolian gerbils, Experimental models

## Abstract

**Objective:**

To compare the incidence and the histopathological aspect of spontaneous and two induced Mongolian gerbils’ models of cholesteatoma: External Auditory Canal (EAC) obliteration model and the Auditory Tube (AT) cauterization model.

**Methods:**

Fifty-four ears of 27 animals were divided into EAC obliteration, AT cauterization, and control groups and histologically assessed for cholesteatoma incidence and classification at intervals of 2, 4, 8, and 16 weeks.

**Results:**

Cholesteatoma was diagnosed in 30 of the 53 ears evaluated with a significantly higher incidence in groups that received some type of intervention (p < 0.0001). It was not possible to histologically distinguish cholesteatomas of the same stage between the study groups.

**Conclusion:**

Although we observed a significant increase in cholesteatoma incidence with the two methods used when compared to the control group, all developed cholesteatomas were apparently identical from a histological point of view.

## Highlights

• Cauterization of the Gerbils’ auditory tube not always induce cholesteatoma formation.
• Gerbils’ models of cholesteatoma may differ from each other and those seen in humans.
• Gerbils’ external auditory canal obliteration induces cholesteatoma in all cases.

## Introduction

Our knowledge of the pathogenesis of cholesteatoma has evolved significantly in the last decades; however, some areas of research remain unelucidated. The existence of congenital cholesteatomas and the formation of cholesteatomas by implantation is incontestable, but are not responsible for all cases.^1^, ^2^ Squamous metaplasia, basal cell hyperplasia and Tympanic Membrane (TM) invagination correspond to other debated theories that, in turn, are unable to explain all the acquired cholesteatomas.^1^, ^3^, ^4^, ^5^ It is believed that a combination of two or more of these theories make possible a better understanding of the pathogenesis of cholesteatoma allowing a more comprehensive and multifactorial conception of the disease.

Although the clinical and experimental evidence suggests that the evolution of this pathology begins with TM retraction followed by loss of self-cleaning properties in this region with accumulation of keratin and consequent formation of cholesteatoma,^4^, ^5^, ^6^ some of these studies report that a series of inflammatory and infectious insults potentially act as crucial determinants in this process.^5^, ^6^, ^7^ Sudoff and Toss^5^ observed the proliferation of keratinocytes within epithelial cones leading to discontinuity areas of the basement membrane of the pars flaccida, especially in the presence of an intense subepithelial inflammatory process, and, therefore, proposed a combination of TM invagination theory with epithelial proliferation.

It is extremely difficult to perform studies in humans to better understand the natural history of this disease. The low incidence in the population, the disease’s slow progression, the difficulty in following-up patients, as well as the difficulty in carrying out histological studies on human temporal bones, have highlighted the necessity of developing animal models for this purpose. In 1981, Chole et al.^8^ reported the spontaneous occurrence of cholesteatoma in Mongolian gerbils and observed that the disease presented similar destructive behavior and histological patterns to that found in humans.

Currently, it is well established that spontaneous aural cholesteatoma in Mongolian gerbils is similar to that induced by obliteration of their External Auditory Canal (EAC).^9^, ^10^, ^11^ However, this model of induction does not reproduce the mechanism of TM invagination initially described by Bezold^3^ and considered by many authors as the best explanation for the pathophysiology of cholesteatoma in humans. To simulate this condition in gerbils, Wolfman and Chole^12^ developed the cauterization model of the Auditory Tube (AT), where they observed the occurrence of pars flaccida retraction and cholesteatoma formation. While some studies point to histopathological similarities between the EAC obliteration and AT cauterization animal models and the clinical form of the disease,^13^, ^14^, ^15^, ^16^ Choufani et al.^17^ have reported differences in the expression of biological markers between these models. Therefore, the pathophysiological mechanisms of cholesteatoma formation by EAC obliteration or AT cauterization in Mongolian gerbils may differ from each other and those observed in humans.

Hence, we aimed to compare the incidence and pattern of induced cholesteatomas and conduct histological comparisons between Mongolian gerbils receiving EAC obliteration and those receiving cauterization of the Auditory Tube (AT) to improve our understanding of the pathogenesis of this disease.

## Methods

Twenty-seven male Mongolian gerbils (*Meriones unguiculatus*), with ages ranging from 102 to 151 days (mean 125 ± 16.3 days) were obtained from an animal house located in the city of Santa Maria. They were kept at the Animal Experimentation Unit (AEU) of the Clinical Hospital of Porto Alegre, with relative air humidity of 40%–60%, controlled temperature (22 ± 2 °C), light cycle of 12 h light/12 h dark, air exhaust system, and received standard feed for the species, as well as water ad libitum. The study was approved by the Ethics Committee on the Use of Animals of the Clinical Hospital of Porto Alegre with de project no. 160463. All study procedures were carried out in accordance with the Brazilian Guidelines for Care and Use of Animals in Teaching or Scientific Research Activities.^18^ We did our best to minimize suffering and provide comfort to the animals throughout the study.

### Cholesteatoma induction procedures

All specimens underwent general inhalation anesthesia with isoflurane (4%–5% concentration induction and maintenance with 2%–3%) and bilateral otoendoscopy prior to the cholesteatoma induction procedure. Any ears that presented with complete EAC filling by cerumen, external ear malformations, or findings compatible with chronic otitis media with cholesteatoma were excluded from the analysis.

Cholesteatoma was induced by two methods: (1) EAC obliteration and (2) AT cauterization. In the first method, the right EAC cartilage portion of 18 ears from 18 animals was obliterated by surgical ligature with a silk thread (4.0) through a retroauricular incision, as proposed by McGinn et al.,^9^ composing Group 1 (n = 18 ears). In the second method, 9 animals underwent bilateral AT cauterization using a needle-point cautery tip inserted through the soft palate in the midline and directed to the right and left nasopharyngeal walls, following a technique described by Wolfman and Chole,^12^ and were termed Group 2 (n = 18 ears). One of the ears of Group 2 was excluded from the final analysis because it presented cerumen filling the entire EAC in the initial otoendoscopy (n = 17). The left ears from 18 animals of Group 1 made up the control group (n = 18).

### Histological analysis

After the cholesteatoma induction procedure, the animals were sacrificed at the following time intervals: two, four and eight weeks (four animals do Group 1 and control group; and two animals from Group 2); and sixteen weeks (six animals from Group 1 and control group; and three animals from Group 2). The specimens were euthanized with an overdose of isoflurane anesthetic (9%–12%) vaporized in 100% oxygen (O_2_ flow of 0.5 L/min) at a concentration ≥ 5%, supplied by inhalation. After confirmation of death by a veterinarian, otoendoscopy of both ears was performed in all groups. In Group 1, the otoendoscopy allowed to verify EAC integrity of the surgical ligature. Then, the right and left bullae were removed from the animals and fixed in buffered formalin 10% solution for 48 h. At the end of this period, they were decalcified in 10% EDTA (changed every 24 h) until reached an ideal stage where the bone could be pierced with a thin needle with minimal resistance and without breaking (after 7–10 days). After processing, the specimens were embedded in paraffin and sectioned into slices 5–10 μm thick in the axial plane using a microtome. For histological analysis, the most representative sections were selected at the level of the EAC midline, which included the external auditory canal, the tympanic membrane, the promontory, and part of the animal's bulla in the same plane. After fixation, the slides were stained with hematoxylin and eosin and evaluated by an AEU pathologist (blinded to the group to which the ear belonged) using a Nikon® E100 binocular microscope (Tokyo, Japan), at 60× magnification.

We followed the guidelines proposed by Chole et al.^8^ for definition and classification of cholesteatoma into five stages: Stage 1 – Accumulation of keratin in the EAC and/or along the TM, without medial displacement of the same; Stage 2 – Keratin filling the EAC and causing TM medial displacement, without contact with the promontory: Stage 3 – The medially displaced TM touches the promontory of the animal; Stage 4 – Presence of inflammatory material and keratin filling the animal’s bulla; Stage 5 – Presence of extension of the inflammatory process to the intracranial region.

### Statistical analysis

We used previously reported incidence of cholesteatoma in the different groups studied^10^, ^11^, ^12^ and a statistical power of 80% with an alpha error of 0.05, to calculate that the smallest sample size of this study should be 31 ears. To evaluate cholesteatoma formation over time, we decided to allocate at least 4 ears per group for each follow-up period, resulting in a minimum of 16 ears per group (total of 24 animals). Considering a possible loss of 10% in the sample, a total of 27 animals (54 ears) were included in the study. The difference in incidence of cholesteatoma between groups was calculated using the Chi-Square test of homogeneity of proportions.

## Results

Spontaneous or induced aural cholesteatoma was observed in 30 of the 53 ears evaluated (56.6%). In Table 1, the incidence of cholesteatoma is presented according to follow-up period for the different study groups (Table 1).

**Table 1:** Incidence of cholesteatoma according to the study groups and the follow-up period.

Groups	2 weeks	4 weeks	8 weeks	16 weeks	Total
	n/total (%)	n/total (%)	n/total (%)	n/total (%)	n/total (%)
Group 1	4/4 (100)	4/4 (100)	4/4 (100)	6/6 (100)	18/18 (100)
Group 2	1/3 (33.3)	2/4 (50)	3/4 (75)	3/6 (50)	9/17 (52.9)
Control	0/4 (0)	0/4 (0)	1/4 (25)	2/6 (33)	3/18 (16.7)
Total	5/11 (45.4)	6/12 (50)	8/12 (66.6)	9/18 (50)	30/53 (56.6)

Group 1, Ears that underwent obliteration of the external auditory canal; Group 2, Ears that underwent cauterization of the auditory tube; Control, Ears of the control group.

Two weeks after initial evaluation: All four ears in Group 1 showed histological signs suggestive of cholesteatoma, at least in the initial stages. In Group 2, one of the three ears examined was histologically classified with Stage 1 cholesteatoma. None of the four ears of the control group showed histological changes after two weeks.

Four weeks after the initial evaluation: Stage 2 cholesteatoma was identified in four ears in Group 1, two of four ears in Group 2 and none of the four ears in the control group.

Eight weeks after initial evaluation: All four ears in Group 1 presented with Stage 2 cholesteatoma. In Group 2, three of four ears presented with Stage 1 cholesteatoma. One of the four control ears was classified with Stage 1 cholesteatoma.

**Figure 1:**
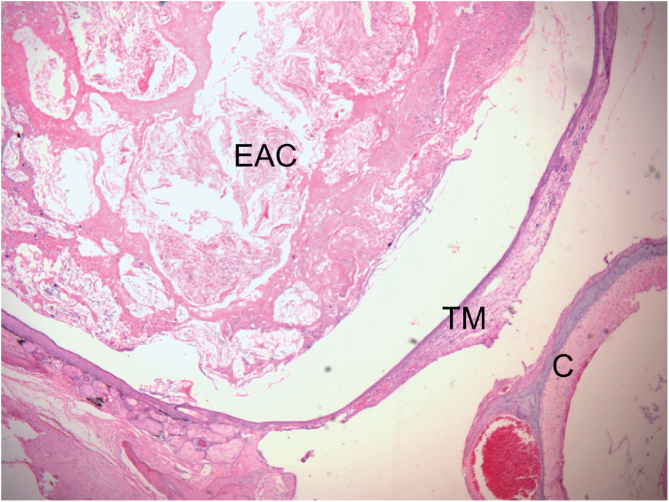
Histological section of right ear stained with H&E, 16 weeks after obliteration of the external auditory canal. External Auditory Canal (EAC) filled with epithelial debris, secretions, and keratin, promoting medial displacement of the Tympanic Membrane (TM), without contact with the Cochlea (C) (Stage 2 cholesteatoma). The blank space between the mass of keratin/epithelial debris occupying the EAC and TM is an artifact incorporated during fixation.

**Figure 2:**
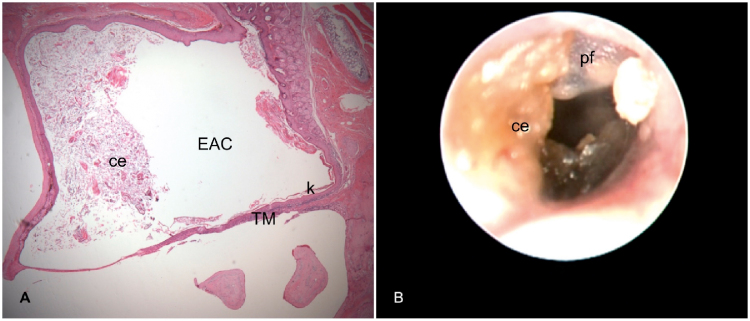
Right ear 16 weeks after cauterization of the auditory tube. A, Histological section with cerumen/epithelial debris (ce) partially occluding the External Auditory Canal (EAC) and Keratin (k) in contact with the Tympanic Membrane (TM) (Stage 1 cholesteatoma). B, Otoendoscopy of the same ear shows in A: Pars Flaccida (pf) thickened and desquamatory; Cerumen/Epithelial debris (ce) in contact with the TM and partially occluding the EAC.

Sixteen weeks after initial evaluation: All six ears of Group 1 developed Stage 2 cholesteatoma (Fig. 1). In Group 2, three of six ears developed cholesteatoma, one with Stage 1 (Fig. 2) and two with Stage 2. Two of the six ears of the control group presented histological signs of cholesteatoma, one with Stage 1 and the other with Stage 2.

No cases of Stages 3, 4, or 5 cholesteatomas were observed in this study. Of the 30 ears that developed cholesteatoma, 11 (36.6%) were classified as Stage 1 and 19 (63.4%) as Stage 2. The incidence of the disease was significantly higher in the groups that received an intervention compared to the control group (Table 2).

**Table 2:** Total incidence of cholesteatoma according to the study groups, regardless of follow-up time.

	Control	Group 1	Group 2	Total	*p*
	n/total (%)	n/total (%)	n/total (%)	n/total (%)	
Normal	15/18 (83,3)	0/18 (0)	8/17 (47,1)	23/53 (43,4)	
Cholesteatoma	3/18 (16,7)	18/18 (100)	9/17 (52,9)	30/53 (56,6)	<0,0001

Group 1, Ears that underwent obliteration of the external auditory canal; Group 2, Ears that underwent cauterization of the auditory tube; Control, Ears of the control group.

**Figure 3:**
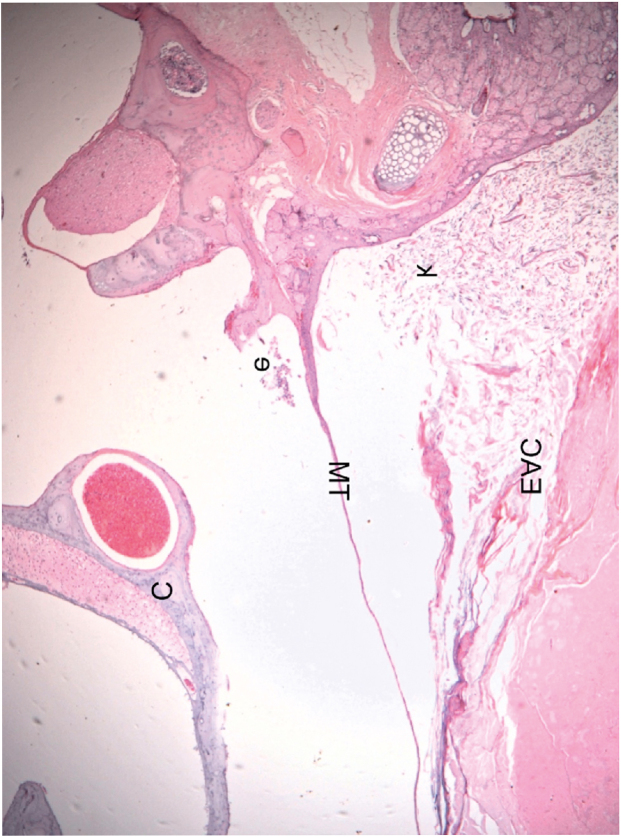
Histological section of an ear 16 weeks after obliteration of the external auditory canal: presence of a few inflammatory cells in the middle ear (C, Cochlea; TM, Tympanic Membrane; EAC, External Auditory Canal; e, Effusion (inflammatory cells); k, Keratin). The blank space between the mass of keratin/epithelial debris occupying the EAC and TM is an artifact incorporated during fixation.

There were no histological differences in the cholesteatomas of the different groups, regardless of the follow-up time. Bone erosion was not observed in the EAC of any ear classified with Stage 2 cholesteatoma. None of the ears presented with Stage 3 disease and none showed bone erosion in the middle ear. A small number of inflammatory cells (polymorphonuclear leucocytes) were also identified inside the bulla of few ears (Fig. 3). We did not observe the formation of retraction pockets either in the pars flaccida or any other TM area in any of the evaluated ears.

## Discussion

In this study, we observed that the incidence of cholesteatoma in Mongolian gerbils after EAC obliteration was significantly higher than that observed after AT cauterization, which in turn was significantly higher than the spontaneous occurrence of the disease in the control group (100%, 52.9% and 16.7%, respectively, *p* < 0.0001), as previously reported in the literature.^9^, ^10^, ^11^ Remembering that Tinling and Chole^11^ reported a 13% incidence of spontaneous cholesteatoma in these animals.

**Figure 4:**
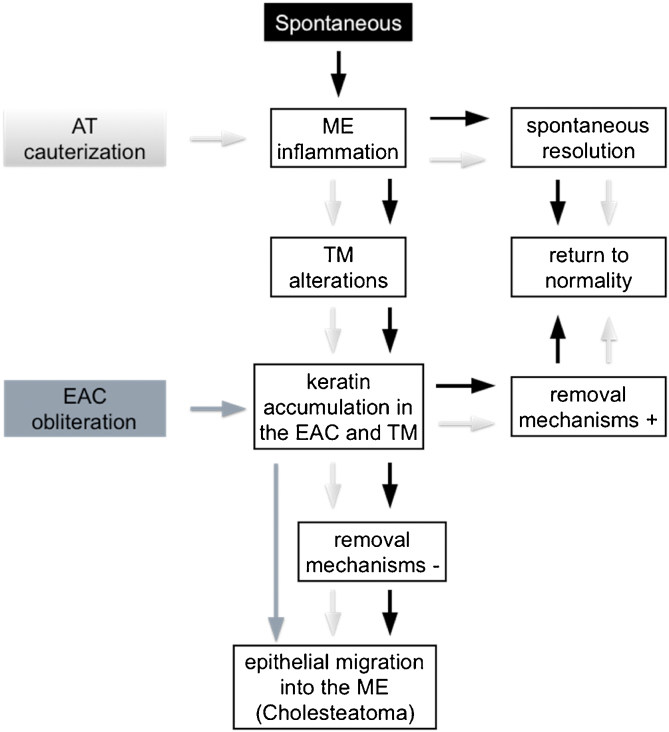
Development of middle ear cholesteatoma in Mongolian gerbils. Spontaneous cholesteatoma (black), AT cauterization model (light gray) and EAC obliteration model (dark gray). (EAC, External Auditory Canal; AT, Auditory Tube; ME, Middle Ear; TM, Tympanic Membrane; +, present; −, absent).

Kim and Chole^10^ described different mechanisms involved in the development of cholesteatoma in Mongolian gerbils by virtue of the induction model used. In 2002, Kim et al.^15^ identified differences in the expression pattern of cytokeratin found in the pars tensa of the TM of these animals between EAC ligation induction methods, AT cauterization, and propylene glycol injection in the middle ear. Based on these reports, it is possible to infer that, theoretically, cholesteatoma is formed through distinct mechanisms in the different Mongolian gerbil cholesteatoma induction models. However, from a practical perspective, we did not find any histological differences in the cholesteatomas formed in the studied groups, regardless of the follow-up period or the method of induction used. These findings are similar to those of Tinling and Chole^13^ who compared the proliferation basal keratinocytes index of cholesteatomas induced by EAC ligation, AT cauterization or both methods combined and reported that it was not possible to distinguish the induction method used through histological analyses performed by a blinded evaluator. Hence, in an attempt to better understand the mechanisms involved in cholesteatoma formation in Mongolian gerbils, we examined three animal models theories, outlined in Fig. 4.

First, the spontaneous occurrence of the disease seems to follow a series of events, as proposed by Tinling and Chole:^11^ initially, although not always visible, an inflammatory process develops in the middle ear, more specifically in the attic region, next to the pars flaccida; this inflammation causes thickening of the TM epithelium due to hyperproliferation of basal keratinocytes, initially in the pars flaccida and then in the pars tensa and fundus of the EAC; failure of keratin and epithelial debris removal mechanisms leads to its progressive accumulation until total occlusion of the EAC and, consequently, medial TM displacement. Based on this theory, there would be at least two ways of inducing and/or accelerating this spontaneous model: (1) Causing an inflammatory reaction in the middle ear; and (2) Favoring the accumulation of keratin and epithelial debris in the EAC. According to some authors,^13^, ^14^ AT cauterization, when successful, is capable of inducing an inflammatory process in the middle ear causing TM structural changes and accumulation of keratin in this region. The ears in which the inflammatory reaction resolves spontaneously and/or in which the self-cleaning mechanisms are fully functional can return to their normal state, as observed in the spontaneous cholesteatoma described by Tinling and Chole.^11^ On the other hand, when a persistent inflammatory process in the middle ear or a failure in the mechanism of epithelial debris removal is present, the progressive accumulation of keratin over the TM and the fundus of EAC could result in the development of cholesteatoma.

The second way of inducing or accelerating the disease in these animals can be achieved by obliterating the EAC, thus invariably provoking the accumulation of epithelial debris and keratin in the EAC and MT. If the surgical suture remains sufficiently coapted, the pressure exerted by the mass of epithelial residues then formed will increase progressively by forcing the TM medially, thus allowing the migration of this content towards the middle ear.

The two induction models mentioned above appear to be capable of causing a state of epithelial hyperproliferation and cholesteatoma formation even in the absence of localized TM retraction.

McGinn et al.^9^ and Kim and Chole^10^ documented cases of Stage 3 cholesteatoma after 12 weeks of EAC obliteration; in contrast, we only observed cases with Stage 1 and Stage 2 cholesteatoma in the present study. According to Aberg et al.,^16^ hyperkeratosis is the primary event in the development of the disease after EAC obliteration. In these cases, the accumulation of keratin evolves until the complete filling of the EAC, pressing the TM medially. Although we reproduced the technique described by McGinn et al.,^9^ some ears evaluated after 16 weeks of follow-up by otoendoscopy presented some degree of EAC suture dehiscence, thus theoretically reducing the pressure in the EAC interior. This fact may have limited our ability to observe disease progression to more advanced stages.

According to Wolfman and Chole,^12^ in some cases, cauterization could not fully result in AT obstruction and, even when successful, the inflammatory process generated in the middle ear in the first days could present spontaneous resolution. For Wilmoth et al.,^14^ TM retractions after this procedure are due to the weakening of its fibrous layer secondary to the activation of metalloproteinases by the chronic inflammatory state generated in the middle ear. An analysis of only the ears that underwent AT cauterization showed an incidence of cholesteatoma of 52.9% after 16 weeks, which was lower than the 75% previously observed by Wolfman and Chole^12^ for the same follow-up period and 100% obtained by Kim and Chole^10^ after 12 weeks of intervention. A higher incidence of the disease could have been observed if we had verified the presence of inflammatory signs in the middle ear a few days after AT cauterization. Considering that the technique used was the same widely disseminated by Wolfmann and Chole,^12^ we chose not to submit the animals to another anesthetic procedure just for this verification in order to minimize their suffering. Therefore, the low incidence observed in the present study may be related to the failure of the procedure to promote a sustained inflammatory reaction in the middle ear of the animals.

Several authors consider the TM invagination theory as the one that best explains the occurrence of cholesteatoma. In line with this thinking, a research group conducted a series of studies on the Contralateral Ear (CLE) of patients with chronic otitis media. In 2008, Costa et al.^19^ evaluated 198 patients with cholesteatoma by otoendoscopy and found abnormalities in 83.3% of CLEs, with 24.8% of patients having severe retractions and 13.3% showing cholesteatoma in the CLE. Rosito et al.^20^ observed that, when present, CLE retractions occurred more commonly in the same TM region where cholesteatoma developed in the main ear. In this way, tubal disfunction appears as one of the main causal factor of cholesteatomas due to damage in the ventilation and consequent negative pressure generated inside the middle ear leading to the formation of TM retraction pockets.

However, according to our findings in the present study, it does not appear that AT cauterization by this method is always able to generate negative pressure in the middle ear long enough to induce TM invagination in experimental models using Mongolian gerbils. In these cases, it could act as a catalyst favoring the appearance of inflammation in the middle ear that promotes the occurrence of TM structural alterations, but not necessarily retraction pockets. Hence, additional measures would be required to replicate the theory of TM retraction in the Mongolian gerbil animal model. Future studies should focus on developing new techniques capable of simulating tubal dysfunction for prolonged periods.

## Conclusion

Both the EAC obliteration model and the AT cauterization model of cholesteatoma increased the incidence of cholesteatoma when compared to spontaneous disease occurrence in Mongolian gerbils. None of the models induced the formation of TM retraction pockets in this study. Although the cholesteatomas originated from different pathophysiological mechanisms, both techniques were apparently identical to each other and to the spontaneous cholesteatoma from a histological point of view. The improvement of these techniques or the development of new experimental models can help in the search for missing links in the pathogenesis of cholesteatoma.

## Funding

Reserch and Events Incentive Fund of Hospital de Clínicas de Porto Alegre (FIPE/HCPA).

## Conflicts of interest

The authors declare no conflicts of interest.

## References

[1] Sadé J., Babiacki A., Pinkus G. (1983). The metaplastic and congenital origin of cholesteatoma. Acta Otolaryngol.

[2] Schroer R. (1958). 2 cases of traumatic cholesteatoma of middle ear. Z Laryngol Rhinol Otol.

[3] Bezold F. (1890). Perforation of Shrapnell’s membrane and occlusion of the tubes: an aetiological study. Arch Otolaryngol.

[4] Sadé J., Avraham S., Brown M. (1981). Atelectasis, retraction pockets and cholesteatoma. Acta Otolaryngol.

[5] Sudhoff H., Tos M. (2000). Pathogenesis of attic cholesteatoma: clinical and immunohistochemical support for combination of retraction theory and proliferation theory. Am J Otol.

[6] Sudhoff H., Tos M. (2007). Pathogenesis of sinus cholesteatoma. Eur Arch Otorhinolaryngol.

[7] Juhn S.K., Jung M.K., Hoffman M.D., Drew B.R., Preciado D.A., Sausen N.J. (2008). The role of inflammatory mediators in the pathogenesis of otitis media and sequelae. Clin Exp Otorhinolaryngol.

[8] Chole R.A., Henry K.R., McGinn M.D. (1981). Cholesteatoma: spontaneous occurrence in the mongolian gerbil Meriones unguiculatus. Am J Otol.

[9] McGinn M.D., Chole R.A., Henry K.R. (1982). Cholesteatoma. Experimental induction in the Mongolian Gerbil, Meriones Unguiculatus. Acta Otolaryngol.

[10] Kim H.J., Chole R.A. (1998). Experimental models of aural cholesteatomas in Mongolian gerbils. An Otol Rhinol Laryngol.

[11] Tinling S.P., Chole R.A. (2006). Gerbilline cholesteatoma development. Part II: temporal histopathologic changes in the tympanic membrane and middle ear. Otolaryngol Head Neck Surg.

[12] Wolfman D.E., Chole R.A. (1986). Experimental Retraction Pocket Cholesteatoma. Ann Otol Rhinol Laryngol.

[13] Tinling S.P., Chole R.A. (2006). Gerbilline cholesteatoma development Part III. Increased proliferation index of basal keratinocytes of the tympanic membrane and external ear canal. Otolaryngol Head Neck Surg.

[14] Wilmoth J.G., Schultz G.S., Antonelli P.J. (2003). Matrix metalloproteinases in a gerbil cholesteatoma model. Otolaryngol Head Neck Surg.

[15] Kim H.J., Tinling S.P., Chole R.A. (2002). Increased proliferation and migration of epithelium in advancing experimental cholesteatomas. Otol Neurotol.

[16] Aberg B., Edstrom S., Bagger-Sjoback D., Kindblom L.G. (1993). Morphologic development of experimental cholesteatoma. Arch Otolaryngol Head Neck Surg.

[17] Choufani G., Roper N., Delbrouck C., Hassid S., Gabius H.J. (2007). Animal model for cholesteatoma induced in the gerbil: will the profiles of differentiation/growth-regulatory markers be similar to the clinical situation?. Laryngoscope.

[18] mctic.gov.br [Internet]. Brasília (DF). [Normative Resoluption nº 30. 2016, February 2nd. Brazilian Guideline for the Care and Use of Animals in Teaching or Scientific Research Activities – DBCA]. Avaiable from: http://concea.mctic.gov.br.

[19] Costa S.S., Rosito L.P., Dornelles C., Sperling N. (2008). The contralateral ear in chronic otitis media: a series of 500 patients. Arch Otolaryngol Head Neck Surg.

[20] Rosito L.P.S., Sperling N., Teixeira A.R., Selaimen F.A., Costa S.S. (2018). The role of tympanic membrane retractions in cholesteatoma pathogenesis. Biomed Res Int.

